# The learning curve of sting method for endoscopic injection treatment of vesicoureteral reflux

**DOI:** 10.1590/S1677-5538.IBJU.2017.0465

**Published:** 2018

**Authors:** Ayhan Dalkiliç, Göksel Bayar, Hasan Demirkan, Kaya Horasanli

**Affiliations:** 1 Department of Urology, Sisli Hamidiye Etfal Training and Research Hospital, Istanbul, Turkey; 2 Department of Urology, Martyr Prof. Dr. Ilhan Varank Sancaktepe Training and Research Hospital, Turkey; 3 Department of Pediatric Urology, Sisli Hamidiye Etfal Training and Research Hospital, Istanbul, Turkey

**Keywords:** Learning Curve, Endoscopy, Vesico-Ureteral Reflux

## Abstract

**Objective:**

To identify how many endoscopic injection (EI) procedures, STING method, must be performed before reaching an ideal success rate when simulation training has not been received.

**Materials and Methods:**

The EI procedures performed by two pediatric urology fellows were investigated. The study excluded patients without primary VUR and those with previous EI or ureteroneocystostomy, lower urinary tract dysfunction, and/or duplicate ureters. The EIs used dextranomer hyaluronate and the STING method, as described by O’Donnell and Puri. Groups number was determined by multiple statistical trials. Statistically significance differences were achieved with one combination that had 35 EI procedures each and with 3 different combination of patients, having 12, 24, and 36 patients, respectively. Therefore, groups were established 12 patients. The first fellow performed 54 EIs, and the second performed 51. Therefore, each of the first fellow's three groups contained 18 EI procedures, and each of the second fellow's 17.

**Results:**

The study included 72 patients and 105 ureter units. When the data from both fellows were combined, each of the three groups contained 35 procedures. For the first fellow, the success rates in the first, second, and third groups were 38.3%, 66.6%, and 83.3% (*p* = 0.02), respectively, and for the second fellow, the success rates were 41.2%, 64.7%, and 82.3% (*p* = 0.045), respectively. The increased success rates for both fellows were very similar.

**Conclusions:**

An acceptable rate of success for EI may be reached after about 20 procedures and a high success rate after about 35-40 procedures.

## INTRODUCTION

Vesicoureteral reflux (VUR) is very common, having an incidence of about 1% in all children (1). VUR is one of the causes of childhood hypertension and chronic renal failure (CRF) (2). In the approach to patients with VUR, the primary aim is not to correct the VUR but to prevent febrile urinary tract infections (UTIs) and CRF due to the formation of scar tissue caused by UTIs (3). For the majority of patients, VUR resolves without requiring any intervention; however, some patients may require surgical treatment, endoscopic injection (EI), or ureteroneocystostomy (4).

The success of an EI procedure having one or more injections is about 85%, and the factors that reduce the success rate include the presence of a high degree of reflux, duplicate systems, and neuropathic bladder (5). In addition to these patient-linked factors, the success of the EI procedure is affected by the operator's experience (6, 7). As EI is an endoscopic procedure performed by a single operator using a single device and needle, training can be difficult (8). Therefore, ex-vivo and computer-based simulation programs have been used to increase the success rates for EI procedures (8, 9). Those without this training should conduct EI procedures only under expert observation until they have fully learned the procedure.

We hypothesized that the learning curve for EI is longer than has been expected, especially in the absence of ex-vivo or simulation training. The present study aimed to identify how many procedures were required for an operator who had had no ex-vivo or simulation training to reach an acceptable rate of success and an ideal rate of success.

## MATERIALS AND METHODS

This study retrospectively evaluated 91 patients who had undergone an EI due to VUR between 2013 and 2016 at our clinic. The study excluded those patients who had not had primary VUR; those who had had previous surgical intervention or EIs for VUR; those who had duplex ureters, CRF, or bladder bowel dysfunction (BBD); and those who had not had at least a 1-year follow up. All EI procedures were completed by the same 2 pediatric urology fellows, each of whom had a 6-month rotation. During their main training periods in urology and pediatric surgery, each fellow had completed three EI procedures under supervision.

Before surgery, a medical history was taken from each patient, and all symptoms were described. In addition, a renal bladder ultrasound (RBUS), a voiding cystourethrography (VCUG), static renal scintigraphy imaging, and creatinine measurements were taken. For those without toilet training, EI was conducted in the presence of the following indications: breakthrough infections (febrile UTI in spite of continue antibiotic prophylaxis), and/or formation of new kidney scar tissue. For those who were toilet trained, EI was conducted in the case of febrile UTI or formation of new scar tissue. All EIs were performed using the STING method described by O’Donnell and Puri (10) and using dextranomer hyaluronic acid (Dx/HA) (Dexell-Vur^®^; Turkey). The needle was placed under the bladder mucosa about 3 mm below the affected ureteral orifice, at the 6 o'clock position, and the Dexell was injected inside the lumen until adequate mound morphology was attained. Before performing the first EI, each fellow watched at least 15 procedures being performed by a supervisor. Supervisor has not intervened to any case directly while the fellows were performing EI, because both fellows are specialist and have authority for EI. The fellows always decided the amount of material to inject and manipulated the needle in the submucosa. Three months after the EI procedure, each patient underwent a control VCUG. Even if no reflux was seen on this VCUG, each patient was followed up for at least 1 year in terms of infection. Success was defined as no reflux being seen on VCUG, no manifest hydronephrosis being seen on urinary ultrasonography both in the third month and in the first-year control and no new scar on renal scintigraphy was observed in the first-year control. Second EIs, due to unsuccessful, were performed by pediatric urology specialist, so these EIs were not included in the analysis, but first EIs were included to analyze as unsuccessful procedure.

To grade reflux, the international reflux degree system was used. Grades 1, 2, and 3 were classified as low, and Grades 4 and 5 were classified as high. Each patient's age, gender, side and degree and grade of reflux, toilet-training status, dilatation on RBUS, and scar presence on renal scintigraphy were recorded.

The study analyzed 72 patients and 105 EI procedures after exclusion. To identify the ideal number of patients that indicated statistical significance, we placed the patients in a different number of groups. For instance, the 105 EI procedures were placed in groups of 5, 7, 10, 15, 21, and 35, and the 72 patients were placed in groups of 6, 9, 12, 18, 24, and 36. Statistically significance differences were achieved with one combination that had 35 EI procedures each and with 3 different combination of patients, having 12, 24, and 36 patients, respectively. Therefore, for each fellow (Fellow 1 and Fellow 2), 3 groups were established (Groups 1, 2, and 3), each of which contained 12 patients. For each fellow, the number of EIs performed was divided into 3 groups based on chronological order, with each group containing an equal number of cases. Fellow 1 performed 54 procedures, and Fellow 2 performed 51. Therefore, each of Fellow 1's 3 groups contained 18 procedures, and each of Fellow 2's 3 groups contained 17. This means that the sum of the procedures in Fellow 1's Group 1 and Fellow 2's Group 1 equaled 35, the sum of the procedures in their Group 2s equaled 35, and the sum of the procedures in their Group 3s equaled 35. All patients were evaluated together in terms of demographic and basic information, operation success, and the amount of material injected. In addition, success rates and the amount of material injected were calculated separately for each fellow.

Statistical analysis used the Kruskal-Wallis, one-way analysis of variance (ANOVA), and chi-square tests, and *p* values less than 0.05 were accepted as statistically significant. All procedures were conducted in accordance with the ethical standards of the institutional and/or national research committees and with the 1964 Helsinki Declaration and its later amendments or with comparable ethical standards. The authors conformed ethic rules of Committee on Publication Ethics and the International Committee of Medical Journal Editors. Human Research Ethics Committee and Institutional Review Board approvals were obtained from Ankara Training and Research Hospital committees. Informed consent was not obtained due to the retrospective nature of the study.

## RESULTS

The study included 72 patients and 105 ureter units. The EIs were unilateral in 39 patients and bilateral in 33 patients (66 ureter units). Median age was 7 (1-15) years, there were 22 males (30.5%) and 50 females (69.5%), and there were 77 (73.3%) low-grade reflux ureters and 28 (26.7%) high-grade ones. Of the 105 procedures, 66 (62.8%) were successful. Of the 39 unsuccessful ureter units, 34 received a second injection. Of these 34 second injections, 25 were successful. Each of the 9 ureters that experienced failure of the second injection and the 5 ureters that experienced failure of the first injection and did not undergo a second one received a ureteroneocystostomy procedure. The median follow-up time was 2.5 years (1-4). No major complications (ureterovesical junction stenosis, sepsis, etc.) were observed.

Age, gender, presence of renal scarring, grade, degree of reflux (low or high), side (right or left), laterality (uni-or bilateral) and toilet-training status were similar in all three groups (Table-1). Grade of reflux were similar according to groups for each fellow (Table-2).

**Table 1:** Descriptive analyze of the studied population (*: p value is significant under 0.05).

	All	1st Group	2nd Group	3rd Group	p value
Patient/EI number (n)	72/105	23/35	25/35	23/35	
Female/Male	74/31	23/12	22/13	29/6	0.121
Median age (years) (min-max)	7 (1-15)	7.5(2-15)	6(1-15)	7(2-14)	0.807
Right/Left	42/63	14/21	15/20	13/22	0.935
Uni/Bilateral	39/66	13/22	15/20	11/24	0.702
Low/High Grade	77/28	27/8	24/11	26/9	0.675
Grade 1/2/3/4/5	5/24/48/13/15	1/10/16/3/5	2/7/15/5/6	2/7/17/5/4	0.971
Renal scar presence (no/minimal/extensive)	50/28/27	17/8/10	18/6/11	15/14/6	0.087
No toilet trained /trained patients nu	30/42	11/13	9/15	10/14	0.842

**Table 2:** Number of ureter units according to grade of reflux for each fellow.

	1st Fellow				2nd Fellow			
Grade	1st Group	2nd Group	3rd Group	P value	1st Group	2nd Group	3rd Group	P value
1	0	1	2	0.706	1	1	0	0.915
2	5	3	3		5	4	4	
3	8	8	8		8	7	9	
4	2	2	4		1	3	1	
5	3	4	1		2	2	3	

**Figure 1:**
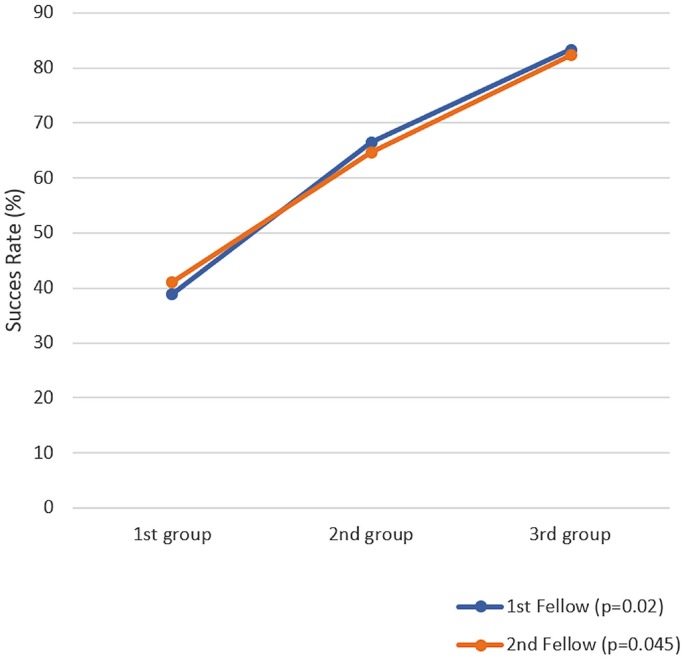
Variation in EI success rates of all patients according to groups.

**Figure 2:**
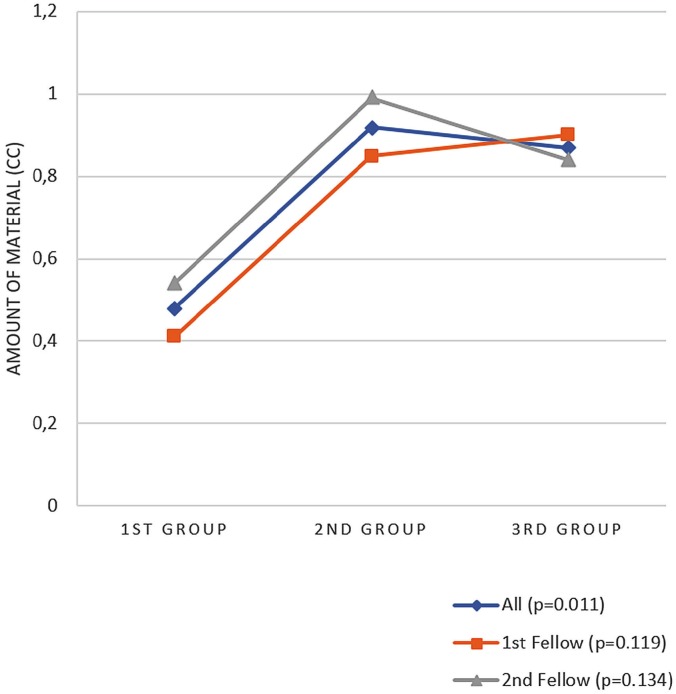
Variation in mean material injected per ureter unit according to groups.

Success rates for the EI procedures clearly differed among groups. In the first group, the success rate was 40%; in the second group, it was 65.7%; and in the third group, it was 82.8%. The difference among these groups was statistically significant (*p* = 0.001) (Figure-1). The mean amount of material used for an EI was 0.48 cc in the first group, 0.92 cc in second, and 0.87 cc in the third (*p* = 0.011) (Figure-2). Post-hoc analysis showed that the mean amount of material used in the first group was significantly lower than the mean used in the second and third groups, which did not differ significantly from each other.

The total success rates for Fellow 1 and Fellow 2 were 62.9% and 62.7%, respectively. The difference between these two rates was not statistically significant. When the data for each fellow were analyzed by group, the success rates for Fellow 1 were 38.3%, 66.6%, and 83.3% (*p* = 0.02), and those for Fellow 2 were 41.2%, 64.7%, and 82.3% (*p* = 0.045) (Figure-1). For both fellows, the amount of material injected was lower in the first group than in the second and third groups (the *p* value for Fellow 1 was 0.119; for Fellow 2, it was 0.134). The differences among groups were not statistically significant, nor was the difference between fellows (Figure-2).

## DISCUSSION

The STING method was first described in 1984 by O’Donnell and Puri (10), and for many years, it was the standard technique used for EI. In 2004, Kirsch et al. described a modified STING technique called the hydrodistension implantation technique (HIT) (11). Four years later, a double HIT method was described by authors from the same clinic (12). Systematic reviews have demonstrated that the HIT method has better outcomes than the STING method; however, long-term results from randomized prospective studies are needed (13). For the sake of consistency, the present study did not include patients on whom the HIT or double HIT methods had been used but included only those on whom the STING method had been used.

Dx/HA is the only molecule with FDA permission to be used in EI, and it has become the gold standard molecule for demonstrating the success of endoscopic treatment of VUR (14). In a study comparing three different injection materials (collagen, polydimethysiloxane, and Dx/HA), Dx/HA's success rate after one injection was found to be clearly superior to that of the other two molecules (15). For EI procedures, our clinic uses Dexell-Vur^®^ containing Dx/HA of 80-120 μm in size. The success of a second injection after a first unsuccessful injection has been found to be 68%, and the success of a third injection after a second unsuccessful injection has been noted as 34% (5). Factors increasing the failure rate include duplicate ureters and BBD (16, 17). In the present study, the learning curve experienced by operators was the only factor affecting EI success; therefore, the study did not include injections performed after the first one, duplicate systems, and patients with BBD.

Studies related to Dx/HA have shown that the success rate after the first injection varies from 67.5-81.5% (5, 18-20). However, in patients in whom reflux is not observed on a postoperative VCUG, VUR is still observed in the long-term at rates from 13-21% (21). As a result, the success rates reported in the literature may be higher than they should be. We performed a VCUG three months after surgery and again one year after surgery, and we believe that evaluating success at the end of one year accurately reflects the success rate.

Kirsch et al. (22) demonstrated that success rates were 60% in the first 20 and 80% in the last 20 on 292 procedures. They concluded that the success rate was directly related to the learning curve. In the present study, the success rates in the first, second, and third groups were 40%, 65.7%, and 82.8%, respectively. Therefore, the success rate in the second group, 65.7%, was close to what Kirsch et al. noted as an acceptable success rate. The clearly higher success rate for the third group showed that the learning curve for the EI procedure was longer than expected (about 20 procedures) and that the success rate approached the ideal only near the end of this learning curve. Considering that both fellows involved in the study had performed fewer than 5 EIs during training, the number of EIs necessary to reach an acceptable success rate can be calculated as about 20. Each fellow performed another 17 or 18 and 17 EI procedures in their second groups; therefore, the success rate increased again after 35-40 EIs, and the ideal rates were obtained for the procedures performed in the third groups.

One factor affecting the success of an EI procedure is the amount of material injected. According to one study, Dx/HA injections of less than 0.8 mL had success rates of 31.8%, and those of 0.8 mL or more had success rates of 78.9% (7). Some studies of Dx/HA have injected mean amounts of material ranging from 0.9-1 cc in all patients, except for those with ureters suffering high-grade reflux, to whom 1.3 cc was administered (18, 23). In the present study, the mean amount of material used was 0.48 cc in the first group, 0.92 cc in the second group, and 0.87 cc in the third group. We believe that the second group used the mean amount of material that is required for an ideal success rate. When the data for both fellows were compared, it was observed that the amount of material injected by both fellows increased significantly from the first group to the second group and that the difference between the second group and the third group plateaued. We have talked with the fellows about mean injection material changing, they said that they were afraid of iatrogenic ureterovesical junction obstruction, so they have thought that we can do re-injection in case of failure but iatrogenic ureterovesical junction failure is a more complicated issue. This may be the explanation of low material amount of first 20 cases. In the first 20 cases, they may have placed the needle into the submucosa fairly close to the ureterovesical line or may not have placed the needle deep enough. After about 20 procedures, they learned to make the injections using accurate placement and depth, both of which are needed to provide sufficient space for the material injected. It was noted that although the success rates differed significantly between the second and third groups, the amount of material injected did not. This supports our belief that learning the correct angle and axis is what increased the success rates. We believe that the fellows learned accurate placement after about 20 procedures but did not learn the correct angle and axis until after about 35-40 procedures.

There are several reasons why the learning curve for EI is longer than has been expected. First, EI is an endoscopic procedure, so learning is based solely on sight. Other factors that make learning difficult are the lack of tactile feedback when inserting the needle and the lack of knowledge regarding limits on the amount of material. While working on their first groups, both fellows may have injected less material because they were concerned about the risk of hydronephrosis. Additionally, we must accept that there is a difference in endoscopic training between urologist and pediatric surgeon. In the study, we have shown EI success is similar between urologist and pediatric surgeon regardless endoscopic training background.

The retrospective nature of this study and the fact that it included data from only two operators may be considered limitations. However, it must be remembered that a learning-curve study that has a prospective research design carries the risk of bias. In the future, studies that include more fellows would provide more accurate data.

## CONCLUSIONS

Operators who have had no ex-vivo or simulation training may obtain acceptable success rates for EI procedures after about 20 procedures. It may be beneficial for operators learning this procedure by performing it to be observed by an experienced operator for the first 20 procedures. In addition, after 35-40 EI procedures, success rates reach high levels.

## References

[1] Sargent MA (2000). What is the normal prevalence of vesicoureteral reflux?. Pediatr Radiol.

[2] Peters CA, Skoog SJ, Arant BS, Copp HL, Elder JS, Hudson RG (2010). Summary of the AUA Guideline on Management of Primary Vesicoureteral Reflux in Children. J Urol.

[3] Tekgül S, Dogan HS, Kocvara R EAU Guidelines On Paediatric Urology 2017.

[4] Fanos V, Cataldi L (2004). Antibiotics or surgery for vesicoureteric reflux in children. Lancet.

[5] Elder JS, Diaz M, Caldamone AA, Cendron M, Greenfield S, Hurwitz R (2006). Endoscopic therapy for vesicoureteral reflux: a meta-analysis. I. Reflux resolution and urinary tract infection. J Urol.

[6] Bennett SD, Foot LM, Abel EJ, Snow BW, Cartwright PC, Devries CR (2010). Is there a learning curve for subureteric injection of dextranomer/hyaluronic acid in the treatment of vesicoureteral reflux?. J Pediatr Urol..

[7] Dave S, Lorenzo AJ, Khoury AE, Braga LH, Skeldon SJ, Suoub M (2008). Learning from the learning curve: factors associated with successful endoscopic correction of vesicoureteral reflux using dextranomer/hyaluronic acid copolymer. J Urol.

[8] Soltani T, Hidas G, Kelly MS, Kaplan A, Selby B, Billimek J (2016). Endoscopic correction of vesicoureteral reflux simulator curriculum as an effective teaching tool: Pilot study. J Pediatr Urol.

[9] Bauschard M, Maizels M, Kirsch A, Koyle M, Chaviano T, Liu D (2012). Computer-Enhanced Visual Learning Method to Teach Endoscopic Correction of Vesicoureteral Reflux: An Invitation to Residency Training Programs to Utilize the CEVL Method. Adv Urol.

[10] O'Donnell B, Puri P (1984). Treatment of vesicoureteric reflux by endoscopic injection of Teflon. Br Med J (Clin Res Ed).

[11] Kirsch AJ, Perez-Brayfield M, Smith EA, Scherz HC (2004). The modified sting procedure to correct vesicoureteral reflux: improved results with submucosal implantation within the intramural ureter. J Urol.

[12] Cerwinka WH, Scherz HC, Kirsch AJ (2008). Endoscopic treatment of vesicoureteral reflux with dextranomer/hyaluronic acid in children. Adv Urol..

[13] Yap TL, Chen Y, Nah SA, Ong CC, Jacobsen A, Low Y (2016). STING versus HIT technique of endoscopic treatment for vesicoureteral reflux: A systematic review and meta-analysis. J Pediatr Surg.

[14] Puri P, Kutasy B, Colhoun E, Hunziker M (2012). Single center experience with endoscopic subureteral dextranomer/hyaluronic acid injection as first line treatment in 1,551 children with intermediate and high grade vesicoureteral reflux. J Urol.

[15] Stredele RJ, Dietz HG, Stehr M (2013). Long-term results of endoscopic treatment of vesicoureteral reflux in children: comparison of different bulking agents. J Pediatr Urol.

[16] Läckgren G, Wåhlin N, Sköldenberg E, Nevéus T, Stenberg A (2003). Endoscopic treatment of vesicoureteral reflux with dextranomer/hyaluronic acid copolymer is effective in either double ureters or a small kidney. J Urol.

[17] Van Batavia JP, Nees SN, Fast AM, Combs AJ, Glassberg KI (2014). Outcomes of vesicoureteral reflux in children with nonneurogenic lower urinary tract dysfunction treated with dextranomer/hyaluronic acid copolymer (Deflux). J Pediatr Urol.

[18] Karabacak OR, Yalçinkaya F, Altuğ U, Sertçelik N, Demirel F (2014). Does the modified STING method increase the success rate in the management of moderate or high-grade reflux?. Korean J Urol..

[19] Pogorelić Z, Gudelj K, Budimir D, Todorić J, Jukić M, Furlan D (2016). Comparison of dextranomer/hyaluronic acid based bulking agents in the treatment of vesicoureteral reflux in children: Deflux versus Vurdex. Can J Urol.

[20] Turk A, Selimoglu A, Demir K, Celik O, Saglam E, Tarhan F (2014). Endoscopic treatment of vesicoureteral reflux with polyacrylate polyalcohol copolymer and dextranomer/hyaluronic acid in adults. Int Braz J Urol.

[21] Lee EK, Gatti JM, Demarco RT, Murphy JP (2009). Long-term followup of dextranomer/hyaluronic acid injection for vesicoureteral reflux: late failure warrants continued followup. J Urol.

[22] Kirsch AJ, Perez-Brayfield MR, Scherz HC (2003). Minimally invasive treatment of vesicoureteral reflux with endoscopic injection of dextranomer/hyaluronic acid copolymer: the Children's Hospitals of Atlanta experience. J Urol.

[23] Kocaoglu C (2016). Endoscopic treatment of grades IV and V vesicoureteral reflux with two bulking substances: Dextranomer hyaluronic acid copolymer versus polyacrylate polyalcohol copolymer in children. J Pediatr Surg.

